# Image Ambiguity and Fluency

**DOI:** 10.1371/journal.pone.0074084

**Published:** 2013-09-05

**Authors:** Martina Jakesch, Helmut Leder, Michael Forster

**Affiliations:** Faculty of Psychology, Department of Basic Psychological Research and Research Methods, University of Vienna, Vienna, Austria; McGill University, Canada

## Abstract

Ambiguity is often associated with negative affective responses, and enjoying ambiguity seems restricted to only a few situations, such as experiencing art. Nevertheless, theories of judgment formation, especially the “processing fluency account”, suggest that easy-to-process (non-ambiguous) stimuli are processed faster and are therefore preferred to (ambiguous) stimuli, which are hard to process. In a series of six experiments, we investigated these contrasting approaches by manipulating fluency (presentation duration: 10ms, 50ms, 100ms, 500ms, 1000ms) and testing effects of ambiguity (ambiguous versus non-ambiguous pictures of paintings) on classification performance (Part A; speed and accuracy) and aesthetic appreciation (Part B; liking and interest). As indicated by signal detection analyses, classification accuracy increased with presentation duration (Exp. 1a), but we found no effects of ambiguity on classification speed (Exp. 1b). Fifty percent of the participants were able to successfully classify ambiguous content at a presentation duration of 100 ms, and at 500ms even 75% performed above chance level. Ambiguous artworks were found more interesting (in conditions 50ms to 1000ms) and were preferred over non-ambiguous stimuli at 500ms and 1000ms (Exp. 2a - 2c, 3). Importantly, ambiguous images were nonetheless rated significantly harder to process as non-ambiguous images. These results suggest that ambiguity is an essential ingredient in art appreciation even though or maybe *because* it is harder to process.

## Introduction

In our everyday lives we are often confronted with ambiguous information, coming from manifold sources and present in various sensory domains. At early processing stages, sensory ambiguities challenge the visual system. At higher-order levels of processing, where the sensory percept is no longer ambiguous, equivocal interpretation or multiple solutions characterize objects of cognitive ambiguity. For example, in the domain of fine arts, sensory ambiguities emerge when it is unclear whether a dark area is part of an object or a shadow. Cognitive ambiguities are often found in surrealistic paintings when painted objects are combined into dream-like scenes.

There is something particularly interesting about the responses to ambiguity in art. The visual fine arts are a domain for which ambiguity was not only discussed as an essential feature but also as a source of pleasurable aesthetic experiences [1–3]. However, this seems to contradict those theories about formation of preference that claim that easy-to-process (fluent) stimuli are most preferred [4–7]. Therefore, ambiguous images which are harder to interpret should be preferred less than non-ambiguous counterparts. Using reproductions of surrealistic artworks and modified, manipulated versions the present study aims to investigate which of these contrasting theories explain aesthetic judgments for ambiguous and non-ambiguous artistic images.

**Figure 1:**
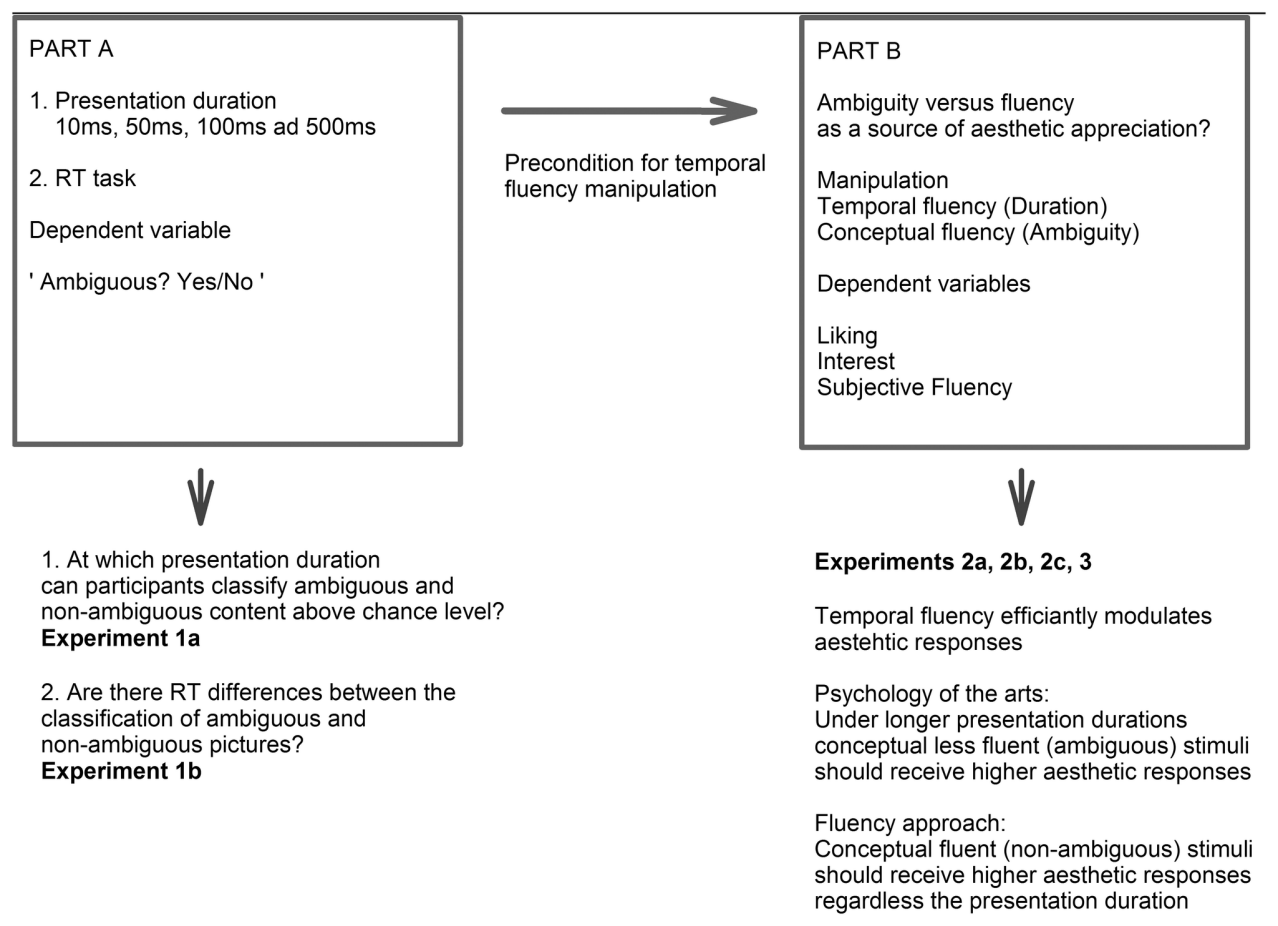
Overview of research questions and experiments.

Research on fluency effects has indicated that the longer something is presented, the more easily it can be processed e.g. [8,9]. However, it is an open issue how long it takes to successfully classify conceptual ambiguity in images. In order to understand how fluency and ambiguity – as features of artworks – affect liking, we first studied how and when ambiguity is processed and classified in surrealistic artworks (Part A). Based on these findings, experiments (Part B) regarding the controversial predictions by current theories in psychology were run: is ambiguity in fine art preferred even though it should be harder to process? Please see Figure 1 for an overview of research questions and experiments.

So far, ambiguity in pictures was mostly studied by means of multi-stable images like Escher figures. Ambiguity has been investigated more frequently in other domains, such as in language processing, social relations and decision making. Therefore, in order to clarify the concept of ambiguity employed in the present studies, different types of ambiguity are discussed in more detail in the following section. Then we provide a short review regarding responses to ambiguous stimuli in classification and appreciation.

### Types of ambiguity

#### Sensory ambiguity

From a vision science perspective it can be argued that the light pattern falling on the retina is generally ambiguous – see 10 for a review – and therefore a challenge for the visual system [11]. Such sensory ambiguous input is encoded by the brain by comparing the input with internal representations. The speed of this comparison depends on the percept and its level of ambiguity but also on the readiness of individuals to impose internal representations [12–14]. At an early stage of processing, familiar interpretations are dominant. Prolonged viewing time then enables alternative (unfamiliar) interpretation(s) [14]. Thus, previous experiences foster the resolution of an ambiguous input [15,16].

Multi-stable or reversible images can reveal insights in processes of sensory ambiguity. These images are characterized by one physical percept that can have at least two different visual experiences. These experiences are reversible (“flip” over time) and cannot be fully controlled by observers. Even though it has been shown that people were able to concentrate on one of the two visual experiences and “hold” it to a significant degree, it was not possible to fully eliminate reversals [10]. The authors assumed that perceptual, bottom-up factors like the early initiation of reversal at ~120 ms but also top-down processes like expectations, learning or contextual information modulate the oscillation rate of multi-stable images. Multi-stability and other sensory ambiguities are playfully used in visual arts. Interestingly, everyday life perception and art perception might differ. In a review, Mamassian [17] argued that prior experience or knowledge is used in everyday life settings whereas conventions are used in visual arts to resolve ambiguity. Even though some of these conventions stem from prior everyday life experiences, others differ to a certain extent like color contrast exaggerations or mirror depictions of figures [17].

#### Cognitive ambiguity

Cognitive ambiguity arises when a stable percept elicits only one visual experience but more than one meaning or interpretation. For example, when a word has more than one meaning – either in spelling, in pronunciation, or both e.g. [18,19]. For example, the word “bat” can stand for a sports gear but also for an animal. In such kinds of homonymous words the alternatives in meaning are usually unrelated. In polysemous words, on the other hand, one word is associated with multiple related metaphorical interpretations, like in “twisting the ankle” or “twisting the truth” [20]. In other domains, cognitive ambiguity can arise from ambiguous facial expression e.g. [21,22], from role confusion regarding job position e.g. [23] or from insufficient information in decision making e.g. [24]. To summarize, ambiguity arises based on sensory and/or cognitive processes and in various domains. In the following section responses to various ambiguous stimuli are discussed in respect to different types of ambiguity and our hypotheses.

### Responses to ambiguous stimuli: Classifying ambiguous and non-ambiguous content

Why is detecting and classifying ambiguity relevant? Successfully encoding the environment enables organisms to plan, trigger, and execute appropriate actions; to respond or react to events, stimuli, or people. For example, in the case of dangerous, threating events resolving ambiguity can even be relevant for survival. If there is not enough, unclear, or ambiguous information, the probability to fail in situations and tasks is increased [25,26]. Thus, Epstein [27] assumed that ambiguity per se would evoke threat and negative emotions. Accordingly, people report lower job satisfaction under higher role ambiguity [23,28], are less efficient [29] and respond with more stress [30]. Further, ambiguity in decision making causes avoidance behavior [31,32]. These examples have in common that ambiguity diminishes successful encoding and processing, and as a consequence, makes it harder to appropriately react to events. All examples are therefore in accordance with a “processing fluency” explanation that hard-to-process situations are negatively connoted, and disliked. Consequently, when confronted with easy to perceive stimuli people feel more confident [33]. This subjective ease of processing is experienced positively and is an important meta-cognitive cue in the formation of human judgments [34,35]. Processing fluency has been shown to influence a variety of responses like reaction times e.g. [4,36], preferences e.g. [4–7], confidence e.g. [33,37], feel of familiarity e.g. [38–40] or truth e.g. [41–43]. Fluency studies often used words/linguistic stimuli [39,40,42,43]. Statements presented in fluent rhyming and prosody were rated as being more true [42,43]. More regular [40] or semantically primed words [8] were rated as more familiar. Because high fluency is associated per definition with high speed and accuracy and low resource demands, it can be assumed that disfluent stimuli are perceived and classified slower than fluent ones [4,36]. Hence, recognizing and processing ambiguous stimuli should take longer. Interestingly, this assumption was supported only for specific tasks. In visual lexical decision tasks (classification as a word or a non-word), several studies found faster reaction times and faster word recognition for ambiguous words (isolated, without context) than for unambiguous words e.g. [44–46]. This “ambiguity advantage effect” was found for polysemous (more than one sense) but not for homonymous words [44,45]. It has been hypothesized that the advantage of ambiguous words might be due to their multiple and rich representations [45,47]. Nevertheless, when a sematic classification task (interpretation of meaning e.g. are two words related?) was used, the reversed pattern was found. This “ambiguity disadvantage effect” – slower responses for ambiguous words – was attributed to the more demanding meaning activation process [48–50] or, alternatively, to the decision making process during semantic tasks [49]. Thus, in lexical decision tasks multiple and rich representations [45,47] of ambiguous words might enhance perceptual and cognitive fluency causing their fast classification. However, when semantic classifications are required, the more demanding process of meaning activation for ambiguous stimuli reduces the classification speed. Consequently, the fluency account would predict that disfluent, ambiguous stimuli are processed slower than fluent, non-ambiguous stimuli [4,36].

What can be predicted for perceiving and classifying ambiguity in images? The multi-stable images used in most previous studies are not suitable to measure classification performance due to their temporally reversal nature. Therefore, in the present studies, we used reproductions of Rene Magritte artworks, always in an ambiguous, and carefully produced non-ambiguous version. Due to the nature of surrealism [51], in Magritte’s paintings semantically unrelated objects are placed in a scene context (semantic violations) or represent syntactic violations. Surrealistic art therefore is similar to metaphorical ambiguity in language [52] and comparable with those real-world scenes used to test object-scene inconsistencies. In this line of research, similar terms as in language processing are used: Inconsistencies were created by inserting a semantically implausible object in a scene context (semantic violation) or by changing the configuration of a scene structure (syntactic violations) like scene-related objects that are moved to unlikely places or floating in the air e.g. [53]. Studies employing eye-movements like those of Loftus and Mackworth [54], indicated that semantically not fitting objects were fixated immediately after stimulus onset and also were fixated longer than other, consistent, objects e.g. [55–57]. According to such a “semantic pop-out” in scenes containing semantic violations, ambiguous artworks might be classified faster than their non-ambiguous counterparts. However, according to the fluency account a reversed pattern would be predicted: Ambiguous artworks should demand more cognitive resources to be interpreted in an (individually) satisfying way than non-ambiguous versions, resulting in slower processing. Thus, it seems that responses to ambiguity are often negative, especially in the case of sensory ambiguity, in social situations or decision-making. Nevertheless, there are some examples for exceptions, where responses to ambiguity are definitely positive.

### The present study

We present two series of experiments (Parts A and B) in which cognitive ambiguity was varied by using surrealistic artworks by Rene Magritte and non-ambiguous control artworks. Part A (“Classifying ambiguous and non-ambiguous content”) is concerned with the classification performance for ambiguous and non-ambiguous pictures (Experiment 1a and 1b). Part B (Appreciation of Ambiguity) investigates the relationship between ambiguity/fluency and aesthetic judgments (liking, interest).

#### Part A: Classifying ambiguous and non-ambiguous content

In Part A we investigate the participants’ performance in successfully classifying ambiguous or non-ambiguous picture content (Experiment 1a) by varying four presentation durations (10ms, 50ms, 100ms, 500ms). The comparison of different times reveals, which presentation duration is sufficient to result in an above chance classification performance. In Experiment 1b we test whether similar results are obtained, when differences in reaction times (RT) instead of differences in presentation duration are analyzed. We expected increasing sensitivity values over the presentation durations (*10ms < 50ms < 100ms < 500ms*). The presentation durations (10ms, 50ms, 100ms, 500ms) were chosen with respect to previous findings. Short durations had been shown to be sufficient for a successful classification (above chance level) of objects in scenes or of scenes themselves. Categorizing photographs on a semantic dimensions (is it an animal or a vehicle?) presented for 20ms resulted in 94% correct responses [73,74]. Above chance performance was found for 27ms presented masked stimuli – even under dual-task conditions [75]. Augustin, Leder, Hutzler, and Carbon [76] showed that the similarity of artworks’ contents can be successfully processed already at 10ms presentation duration. Thus, ten milliseconds were chosen as lower bound. In contrast to shorter presentation durations, participants started to report more semantic categories of viewed scenes between 40 and 67 ms [77]. Fifty milliseconds therefore were used as second presentation duration. The third level, one hundred milliseconds, was chosen according to Fei-Fei et al. [77] and Reber et al. [9] studies. The longest presentation duration of five hundred milliseconds is commonly used as baseline duration, as within this time most of natural scene content is perceived [78–80] and opposed to the shorter durations, a few saccades can be made [77]. The chosen presentation durations will be further used in Part B as manipulation of fluency e.g. [9].

A second question concerned the time needed to detect and to report ambiguity. Experiment 1b was planned to investigate reaction time (RT) differences for ambiguous versus non-ambiguous pictures. We expected that ambiguous pictures are either classified slower than non-ambiguous pictures, based on findings in language research [48,50] and the fluency account [4,36] or faster due to the studies of “semantic pop-out” in scenes containing semantic violations e.g. [55–57].

#### Part B: Appreciation of ambiguity

In Part B, we studied the effects of ambiguity and fluency on aesthetic appreciation (liking, interest). We decided to measure liking to compare previous fluency findings with our data and interest based on the art-historical statements, that simple art with an obvious message appears to be uninteresting [2,60–62]. Interest is characterized by two dimensions /appraisals: novelty/complexity and coping potential [81–86]. According to Silvia’s appraisal theory, objects or events are interesting when unfamiliar, new and complex but comprehensible [82]. This appraisal structure overlaps with one of Csikszentmihalyi’s [69] elements of optimal experience on page 49: “A challenging activity that requires skills”. Further, felt fluency (subjective experience of fluency) was measured as control for the fluency manipulations similar to previous studies [8,36]. Two fluency manipulations were chosen: a) *duration* manipulation [8,9] and b) *ambiguity* as conceptual fluency manipulation (ambiguous versus non-ambiguous pictures). The *duration* manipulation represents a “temporal perceptual” fluency manipulation whereas *ambiguity* represents a higher-order “conceptual” fluency [34]. We assume that effects based on the perceptual fluency manipulation (*duration*) show the proposed positive influence on aesthetic judgments, namely that aesthetic judgments for ambiguous and non-ambiguous judgments increase with the presentation duration. However, in the case of conceptual fluency (*ambiguity*), we expect contrary results modulated by the duration manipulation. Whereas the conceptual fluency manipulation might not influence the aesthetic judgments under short presentation durations, it should be a more prominent cue at longer presentation durations as previous results suggest that semantic processing starts between 40 and 67ms [77]. Bearing in mind that in this case real-world scenes were used (which we are exposed to more frequently than to art), we expect to find effects of *ambiguity* under longer presentation durations (100ms, 500ms). According to the fluency account, conceptually fluent pictures (non-ambiguous ones) should receive higher aesthetic judgments whereas the reversed pattern should be found when the conceptual fluency is overwritten by other higher-order processes [1–3,72,87,88].

## General Method

All four experiments reported here were conducted in respect to the Declaration of Helsinki (revised 1983) and guidelines of the Faculty of Psychology, University of Vienna. According to the Austrian Universities Act 2002 (UG2002) which held at the time the experiments were carried out, only medical universities were required to appoint ethics committees for clinical tests, application of medical methods, and applied medical research. Furthermore, data were collected anonymously and no harming procedures were used. Therefore, ethical approval was not sought for the execution of this study. Written informed consent was given by all participants. Participants could withdraw at any time during the experiment without further consequences.

### Procedure and apparatus

**Figure 2:**
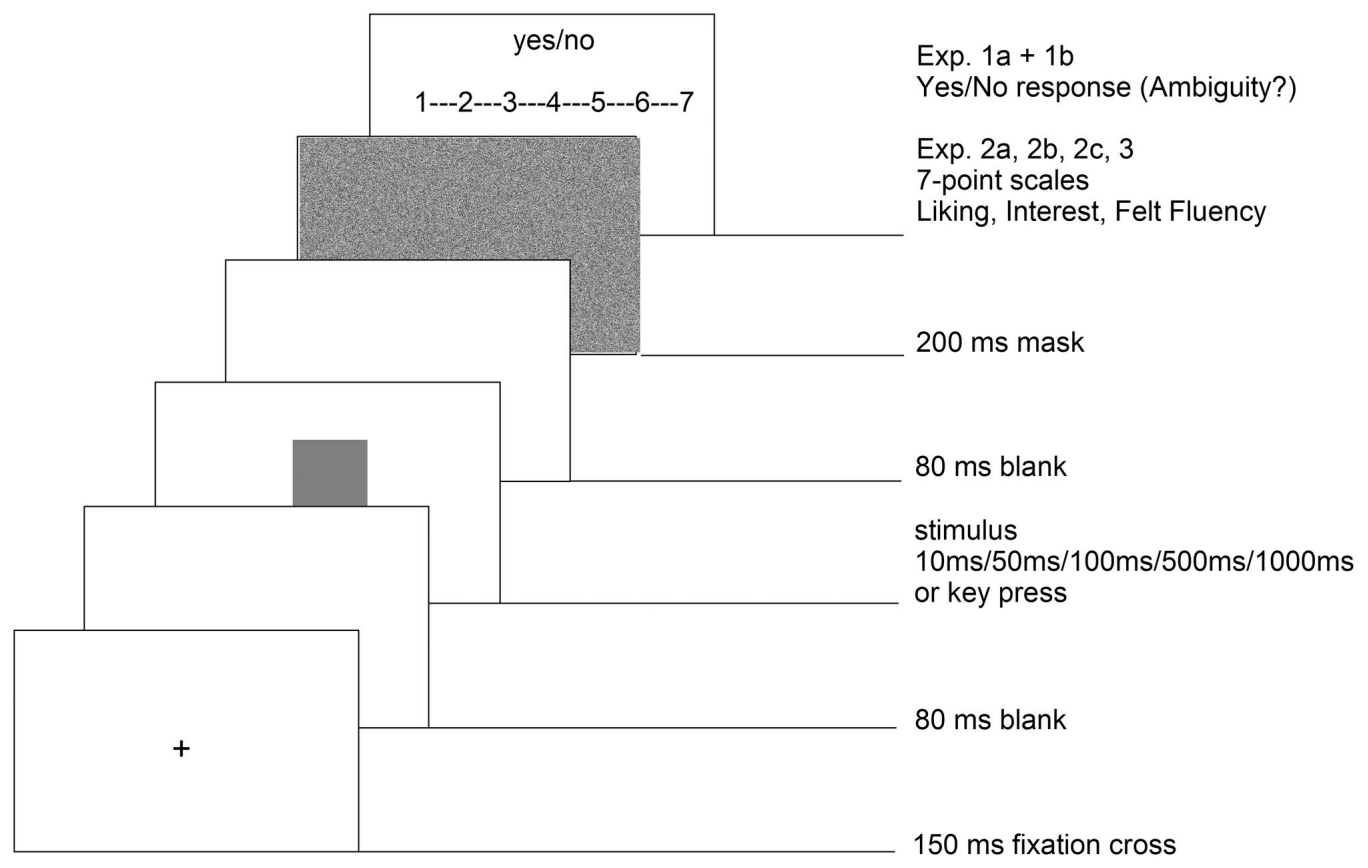
Schematic trial procedure.

Participants were tested individually or in groups of two in a laboratory setting. E-Prime experimental software [89] was used to present the stimuli on screen and record responses (response keys; RTs). Participants sat about 60 cm away from 21-in CRT monitors with 100-Hz refresh rates. Stimuli on the screen subtended 10° × 15° (portrait format) or 15° × 10° (landscape format) of visual angle. Viewing distance (and head position) was kept constant by chin and forehead rests. Visual acuity and color vision (Ishihara color plates) were tested prior to the experiment. In all experiments a similar procedure was used: practice trials were implemented before the main experimental blocks. In the experiments for Part A (1a and 1b) a *Cedrus* Button Box device was used to assure millisecond precision in RTs. The experimental block was divided into two parts with separate practice trials beforehand as the key mapping (yes/no) was balanced across the experiments. In the experiments in Part A, 2 × 8 practice trials and 2 × 22 trials (14 target trials, 8 distractor trials) were run. In Part B, the keyboard was used to record responses on 7-point scales, therefore only one practice block was used in the beginning. After 12 practice trials, the main experimental block started with 56 trials (16 distractor pictures and 36 target trials). In both parts, in a second block all pictures were rated for familiarity on a 7-point scale ranging from 1(not familiar at all) to 7 [very familiar (status before the experiment)]. The trial procedure again remained the same in all experiments (see Figure 2).

A trial started with a fixation cross (150ms), followed by a 80ms blank screen. Then the stimulus was presented: in Experiment 1 either for 10ms, 50ms, 100ms, or 500ms (between subjects); in Experiment 2a 10ms or 50ms (within subjects), 2b 10ms or 100ms (within subjects), 2c 10ms or 500ms (within subjects) and Experiment 3 100ms or 1000ms. Subsequently, a 80ms blank screen was presented followed by a 200ms random noise mask which covered the entire screen. Then the response scale(s) appeared on the screen: in Experiment 1, participants were instructed to respond as fast as possible to the question “Picture ambiguous? Yes or No?” by pressing indicated buttons on a Cedrus Button Box device. The same trial procedure as in Augustin et al. [76] was performed, since similar materials (reproductions of artworks) were used in the study. In the second series of experiments (2a to 2c, 3), the keyboard was used to measure responses from 1 to 7 to the scales liking (How much do you like the current picture? 1: not at all, 7: very much), interest (How interesting is the current picture? 1: not interesting at all, 7: very interesting), and subjective fluency (How easy was it to perceive the current picture? 1: very hard, 7: very easy). The scales were presented in random order. Findings of ERP studies suggest that higher-level visual processing like identification or classification is accomplished after approximately 150ms [80,90–92]. Therefore, the time of post stimulus blank (80ms) and the presented mask (200ms) were chosen to guarantee the processing of these higher-level visual features and similar to the procedure presented in Augustin et al. [76].

### Stimuli

In the present study, 36 pairs of pictures were used plus a set of 32 distractor images. Each pair consisted of an original Magritte painting (ambiguous) and a manipulated (non-ambiguous) version of it. For the manipulated versions, using Adobe Photoshop CS3, we “corrected” the ambiguity to render the painting non-ambiguous in order to provide a fair control condition. This ensured that the original and the manipulated version only differed in terms of ambiguity. For example, objects which were placed in an uncommon way (e.g. the engagement ring around the grand piano in the painting “La main heureuse, 1953”) were either removed (in this example the ring) or modified. Or in “Le Modele rouge, 1947”, where parts of the foot and the toes are combined with brown shoes, the toes were replaced by a “full version” of the shoes. It is important to note, that the picture pairs were balanced across participants: a person saw either the original, ambiguous picture or the manipulated, non-ambiguous one.

In a pre-study, 50 pairs of these artworks were rated according to complexity, mood content, and ambiguity on 7-point scales by 22 participants (Mean age: 24.85 years; 14 female, 8 male). *Ambiguity* was balanced over two experimental versions so that the participants only saw either the original or the corresponding manipulated version of a painting. For the main experiments, we chose picture pairs which significantly varied in their ambiguity, i.e. significantly higher ambiguity ratings for the original picture than for the manipulated version. Further, the picture pairs had to meet the criteria a) medium mood content (mean within the range of 3.0 and 4.0) and b) similar subjective complexity (mean within the range of 3.0 and 4.0). Moreover, 32 distractor images (16 surrealistic = ambiguous; 16 realistic = non-ambiguous) were presented in order to increase the variety of styles that participants were not able to make assumptions concerning the main hypotheses. Responses to distractors were excluded from later analyses. The images measured 269 × 390 pixels (portrait format) or 390 × 269 pixels (landscape format) with a resolution rate of 100 dpi. For a detailed stimulus and distractor list, please see Table S1 (supporting information). Example stimuli and data will be available on request – please contact the corresponding author.

### Part A: Experiment 1a Classification

When are people able to successfully classify ambiguous content of pictures? In this respect, four presentation durations (10ms, 50ms, 100ms, and 500ms) were varied. Signal detection analyses [93] were used to analyze the sensitivity (d Prime *d*’) of classification performance.

#### Participants

Sixty-four psychology students between the ages of 18 and 36 (*M* = 21.90, *SD* = 3.30; 11 male, 53 female) with normal or corrected to normal vision and color vision participated for course credit. None of them had a specific background in art. Written consent was obtained from each participant prior to the experimental session.

#### Design

A 2 (*Ambiguity*: ambiguous versus non-ambiguous) × 4 (*Duration*: 10ms, 50ms, 100ms, 500ms) mixed design was used in the present experiment. The variable *ambiguity* was balanced within subjects. The participants were randomly assigned to one of the four *duration* conditions. As dependent variable, the classification performance in terms of *d’* was measured.

### Results and Discussion Experiment 1a Classification

D primes (*d*’) for each participant were computed, based on hits, false alarms, misses and correct rejections categorization [93]. Extreme values were corrected by using 0.5 ÷ n for rates of 0 and (n - 0.5) ÷ n in the case of rates of 1 as proposed by Macmillan and Kaplan [94] or Stanislaw and Todorov [95]. The *d’* of 10ms, 50ms, 100ms, 500ms were submitted to one sample *t* tests for each duration condition and to a one-way analysis of variance (ANOVA) with duration (10, 50, 100, 500 ms) as between subjects factor. The one sample *t* tests were performed to check the difference of the mean *d’* per *duration* condition to chance level (*d’* = 0). Results showed that the mean *d’* deviated significantly from zero at the specified .05 level (95% CI) in all *duration* conditions: 10ms *t*(15) = 2.13, *p* = .049; Cohen’s d = 1.11, CI of the mean [0.01, 0.78]; 50ms *t*(15) = 5.64, *p* < .001, Cohen’s d = 2.92, CI [0.55, 1.20], 100ms *t*(15) = 8.28, *p* < .001, Cohen’s d = 4.27, CI [0.82,1.40]; and 500ms *t*(15) = 9.11, *p* < .001, Cohen’s d = 4.70, CI [1.05 ,1.69]. In addition, the CIs for each participant-based *d’* [96] were calculated. If the individual CI of each participant’s *d’* includes 0, then the detection performance is not significantly better than chance. The results show that the number of participants with a lower CI bound above 0 increased with longer *duration* conditions: 10ms 1 of 16; 50 ms 5 of 16; 100 ms 8 of 16; and 500 ms 12 of 16, signaling that the longer the stimuli were presented the better the detection performance of the participants became. The mean hit rate (*M*=.61) and mean correct rejection rate (*M*=.68; sampled over all conditions) indicate further that ambiguous images were detected as good as non-ambiguous images were rejected.

**Figure 3:**
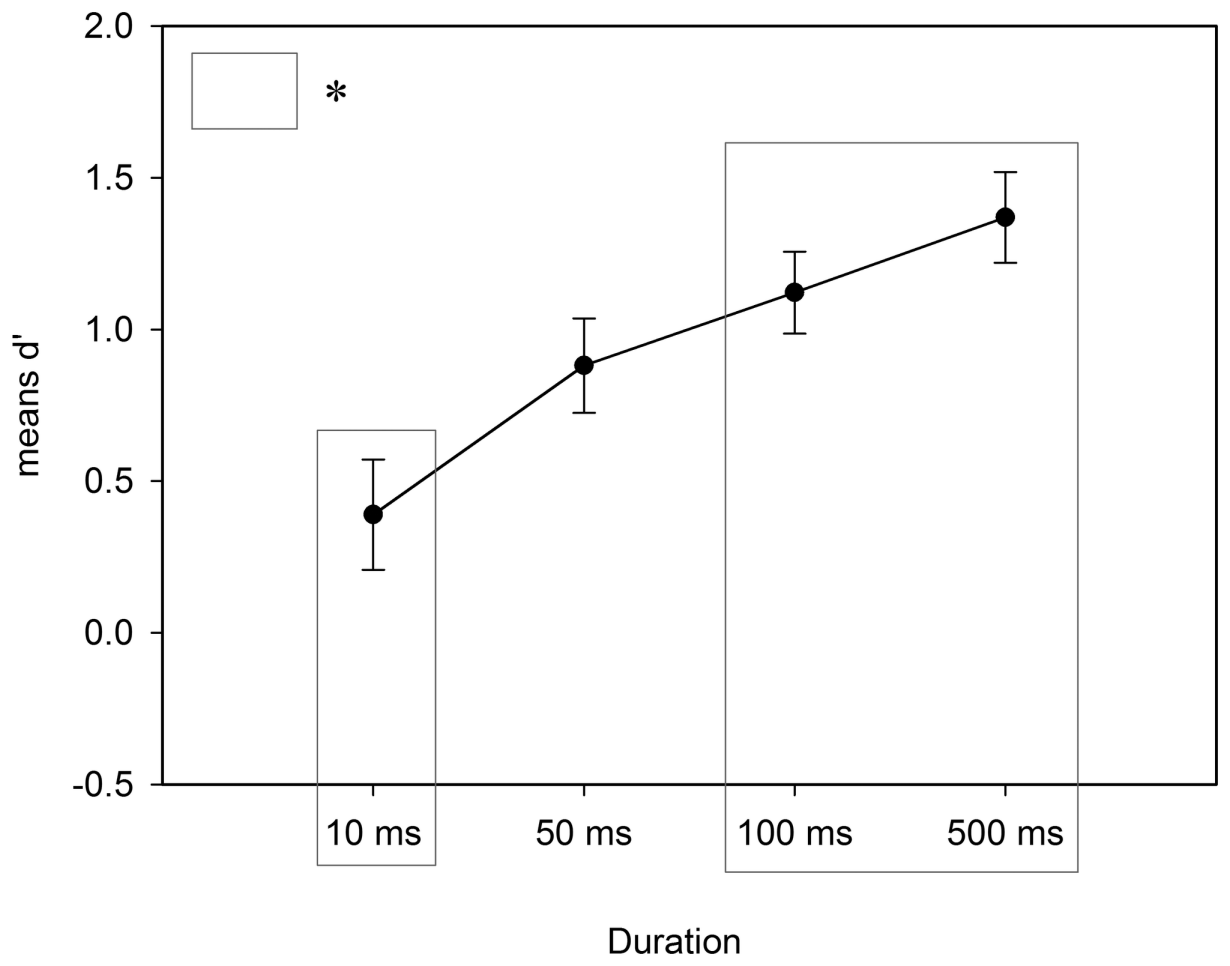
Mean d’ in all duration conditions. The significant difference between the duration conditions 10ms and 100ms / 500ms (see gray boxes) is marked with an asterisk. Error bars represent +/- 1 SE.

Performance differences between the duration conditions were analyzed using an ANOVA with *duration* (10, 50, 100, and 500 ms) as fixed factor and the *d’* as dependent variable. Participants’ classification performance at 10 ms presentation duration was less sensitive (*M* = 0.39) than at 100 ms (*M* = 1.12) and 500 ms (*M* = 1.37) duration resulting in a significant main effect, *F*(3,60) = 7.14, *p* < .01, ηp² = .25. No differences were found among 50, 100, and 500ms (see Figure 3). The familiarity of pictures (measured in a separate block after the main experiment) might influence the performance of classification. Therefore, the number of familiar pictures (pictures which received ratings from 4 to 6 on the 6-point scale) per participant were used as a covariate in an additional ANCOVA. Results showed no significant influence of familiarity on the *d*’, *F*(1,63) = 0.09, *p* = .93, ηp² < .01.

Experiment 1a provided information concerning the duration needed to classify the picture content successfully. Starting at 100ms presentation duration, half of the participants were able to detect the ambiguity above chance level (as indicated by the 95% CI of *d‘*). In Experiment 1b differences in reaction times (RT) for ambiguous and non-ambiguous pictures were tested.

### Part A: Experiment 1b Classification RT

In this experiment reaction times were measured in order to test possible effects of ambiguity in a classification task. According to previous results, ambiguous pictures could either be classified slower (fluency account) or faster (object-scene violations) than non-ambiguous pictures.

#### Participants

Twenty-four psychology students between 18 and 26 years (*M* = 20.79; *SD* = 1.87; 17 female) with normal or corrected to normal vision and color vision participated in the present study. None of them had been tested in Experiment 1a or had a specific background in art. Again, written consent was obtained prior to the experiment.

#### Stimuli

The same twenty-eight picture pairs were used as in Experiment 1a.

#### Design

Design, overall procedure and trial procedure remained the same as in Experiment 1a. In order to test differences in the ability to classify the picture content, a reaction time based task was implemented: instead of a fixed presentation time, after the stimulus onset participants were asked to respond as fast as possible (Picture ambiguous? Yes / No). At a button press the stimulus presentation ended.

### Results and Discussion Experiment 1b

Reaction times (RTs) of correct responses (hits and correct rejections) were analyzed. RT outliers (more than two standard deviations from the mean, 4% excluded) were excluded. The RT means for ambiguous (*M* = 1546.09ms; *SD* = 670.66) and non-ambiguous (*M* = 1587.87ms; *SD* = 719.32) trials were compared in a paired sample *t* test. Results showed no significant difference at the specified .05 level between the responses to ambiguous and non-ambiguous stimuli: *t*(23) = 0.59, *p* = .55, n.s., Cohen’s d = 0.05, 95% CI on the difference between means [-105.80, 189.48].

Surprisingly, there were no differences between the two *ambiguity* conditions. Nevertheless, our findings are in accordance with recently published data by Võ and Henderson [53,97], who did not find reaction time differences for semantic or syntactic violation detection in scenes and consistent control stimuli [53,97]. The mean RTs are comparable with those found for semantically (1964ms) and syntactically (1936ms) consistent and semantically (2067ms) and syntactically (2094ms) inconsistent object-scene combinations [53]. The slightly longer RTs in Võ and Hendersons’ [53] results might be due to higher stimulus complexity. The authors concluded that RT differences for consistent and inconsistent object-scene stimuli might have been due to the saliency of inserted inconsistent objects or spare scene layouts. As we did not manipulate the “inconsistent” ambiguous pictures, the stimuli did not contain such image-based artifacts.

Different from previous fluency results, in Experiment 1b no speed advantage for non-ambiguous pictures was found. However, Oppenheimer [4] noted that equating fluency to reaction times might not be sufficient for all types of fluency and that a subjective feeling of fluency depends on how people anticipate difficulty [8]. He concluded on page 238, that “measuring reaction time ignores this element of the fluency experience entirely” [4]. Therefore, we measured “felt fluency” in addition to aesthetic measures of liking and interest to check our conceptual fluency manipulation (ambiguity).

### Part B: Experiment 2a, 2b and 2c Appreciation of ambiguity

The aim of the current series of experiments was to investigate the relationship between ambiguity/fluency and aesthetic judgments. In order to compare the two fluency manipulations, ambiguous (original) and (manipulated) non-ambiguous Magritte paintings were presented 10ms and 50ms (Experiment 2a), 10ms and 100ms (Experiment 2b) and 10ms and 500ms (Experiment 2c). Experiment 3 (100ms versus 1000ms) was run to compare longer durations. In all experiments, liking, interest and subjective fluency were measured as dependent variables.

#### Stimuli

For the present series of experiments, the set of stimuli used in Experiment 1a and 1b was extended to 36 pairs of pictures in order to provide a higher number of stimuli in the duration conditions (see section Design). Again, each pair consisted of an original Magritte painting (ambiguous) and a manipulated (non-ambiguous) version of it. All stimuli were presented in the same pixel dimensions (269 × 390 pixels) with a resolution rate of 100 dpi.

#### Participants

Sixty (twenty per experiment) participants with normal or corrected to normal vision took part for course credit. The participants’ ages (45 female, 15 male) ranged between 18 and 33 years with a mean age of 22.23 years (SD = 2.67). The procedure was explained prior to the experiment, and written consent was obtained from each participant.

#### Design

A 2 (*ambiguity*) × 2 (*duration*) within subjects design was used. As in Experiment 1a and 1b, *ambiguity* (ambiguous versus non-ambiguous) was manipulated via picture content and balanced across participants. Different to 1a and 1b, *duration* was set as within-subjects factor in order to manipulate fluency as reported in previous studies [9]. Due to the limited number of Magritte paintings which could be manipulated, we had to split the duration conditions. Therefore, three experiments were conducted to compare a) 10ms and 50ms (Experiment 2a), b) 10ms and 100ms (Experiment 2b), and c) 10ms and 500ms (Experiment 2c).

### Results Experiment 2a, 2b and 2c

For each experiment, the data of three dependent variables were submitted to three repeated measures ANOVAs with *ambiguity* (ambiguous versus non-ambiguous) and *duration* (2a: 10ms versus 50ms; 2b: 10ms versus 100ms; 2c: 10ms versus 500ms) as within-subject factors and the dependent measures liking, interest, and felt fluency. In order to compare the findings of the three experiments, the results will be reported in a combined section for each dependent measure.

### Effects on liking

**Figure 4:**
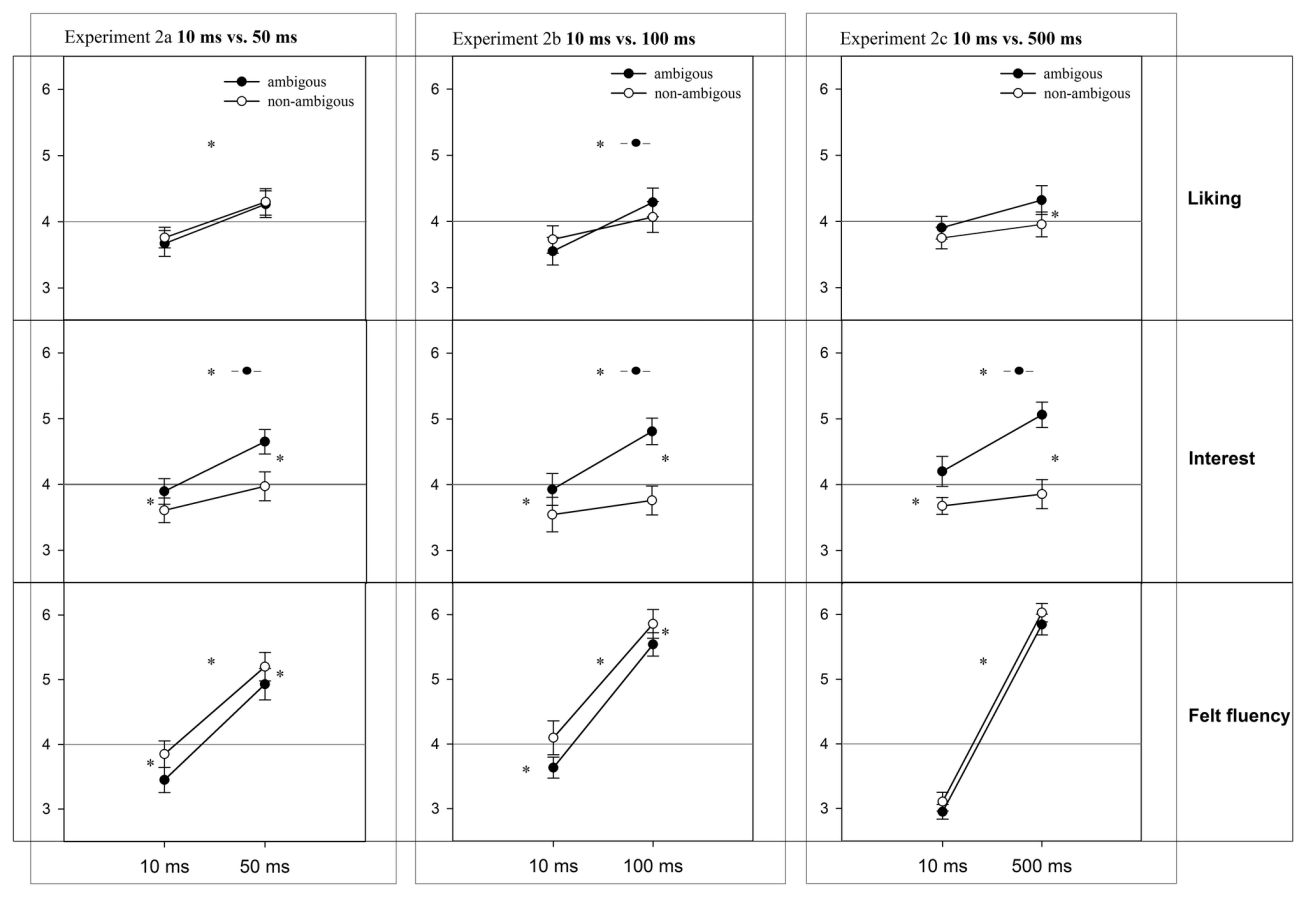
Overview of effects in Experiment 2a, 2b, and 2c.

A significant main effect for *ambiguity* was found in Experiment 2c (10 vs. 500 ms), *F*(1,19) = 5.22, *p* = .03, ηp² = .22; in the Experiment 2a and 2b the ambiguity main effect did not reach significance: 2a [10 vs. 50ms: *F*(1,19) = 0.44, *p* = .50, n.s.], 2b [10 vs. 100ms: *F*(1,19) = 0.028, *p* = .87, n.s.]. The second within-subjects factor *duration* influenced liking significantly in Experiment 2a [10 vs. 50ms: *F*(1,19) = 14.29, *p* = .001, ηp² = .43] and 2b [10 vs. 100ms: *F*(1,19) = 11.62, *p* = .003, ηp² = .38] but not in 2c [10 vs. 500ms: *F*(1,19) = 2.90, *p* = .11, n.s.]. No significant interactions (ambiguity × duration, see Table 1) were found. For an overview of results please see Table 1 and Figure 4.

**Table 1:** Results overview of the ANOVAs’ with repeated measures of Experiment 2a, 2b, 2c and split by the dependent measures liking, interest, and felt fluency (For all three Experiments: df = 1; Error df = 19).

Experiment		2a			2b			2c		
		10/50ms			10/100ms			10/500ms		
Variable		*F*	*p*	ηp²	*F*	*p*	ηp²	*F*	*p*	ηp²
*liking*										
ambiguity		0.44	.05	.01	0.03	.87	.00	5.22	.03*	.22
duration		14.29	.001*	.43	11.62	.003*	.38	2.90	.11	.12
ambiguity×duration		0.08	.76	.01	1.89	.19	.08	1.82	.18	.09
*interest*										
ambiguity		15.40	<.01*	.45	21.75	<.01*	.53	27.72	<.01*	.58
duration		10.84	.004*	.36	14.44	.001*	.43	12.60	.002*	.40
ambiguity×duration		4.51	.04*	.19	12.48	.002*	.40	9.14	.007*	.33
*felt fluency*										
ambiguity		8.42	.009*	.31	10.29	.005*	.35	3.97	.06	.16
duration		61.23	<.01*	.76	375.52	<.01*	.95	306. 41	<.01*	.93
ambiguity×duration		0.75	.34	.04	0.61	.45	.02	0.01	.87	.01

Note: Significant *p*-values at the specified .05 level are marked with an asterisk.

In 2a and 2b, the perceptual fluency manipulation (*duration*) influenced participants liking ratings. No effect for *duration* on liking was found in 2c. Post hoc tests (Bonferroni -adjusted simple main effects) revealed that in 2a ambiguous and non-ambiguous pictures received significantly higher liking ratings at 50ms. In 2b, the main effect of duration is based on significantly higher liking ratings for ambiguous pictures whereas ratings for non-ambiguous pictures did not increase significantly. In 2c, the conceptual fluency manipulation modulated participants’ preferences: at 500ms presentation duration, participants rated the ambiguous pictures as more likeable than non-ambiguous pictures, whereas at shorter durations they did not.

### Effects on interest

In all three experiments, for interest main effects of ambiguity and duration, as well as significant interactions between the two factors, were found (please see Table 1). The data pattern was consistent over all experiments: ambiguous pictures were found significantly more interesting than non-ambiguous pictures under short as well longer durations. Moreover, the mean ratings of interest for ambiguous pictures increased significantly from 10ms to 50ms, 10ms to 100ms, and 10ms to 500ms. This *duration* effect was not found for non-ambiguous pictures (see Figure 4).

### Effects on felt fluency

In all three experiments, strong main effects for *duration* were found (see also Table 1). Participants rated the longer (50ms, 100ms, and 500ms) presented stimuli as more fluent than those presented for 10ms. *Duration*: 2a [10 vs. 50ms: *F*(1,19) = 61.23, *p* < .01, ηp² = .76]; 2b [10 vs. 100ms: *F*(1,19) = 375.52, *p* < .01, ηp² = .95]; and 2c [10 vs. 500ms: *F*(1,19) = 306. 41, *p* < .01, ηp² = .93]. *Ambiguity*, representing a conceptual fluency manipulation, influenced the felt fluency ratings significantly in the expected way in Experiment 2a [10 vs. 50ms: *F*(1,19) = 8.42, *p* = .009, ηp² = .31] and 2b [10 vs. 100ms: *F*(1,19) = 10.29, *p* = .005, ηp² = .35]. Non-ambiguous pictures were significantly rated as being more fluent than ambiguous images under short as well longer presentation durations. This effect was not found in Experiment 2c [10 vs. 500ms: *F*(1,19) = 3.97, *p* = .06, ηp² =.16, trend].

### Discussion Experiment 2a, 2b, and 2c

In accordance with assumptions regarding art, we found that ambiguous paintings can be appreciated. Effects in the favor of ambiguity were found for liking judgments at 500ms and interest judgments from 50 to 500ms. The findings are in accordance with optimal challenges that can be experienced as self-rewarding leading to a positive experience and satisfaction [68]. Ramachandran and Hirstein [52] similarly stated on page 30 that “it is as though an object discovered after a struggle is more pleasing than one that is instantly obvious”. This mechanism explains why our participants rated ambiguous pictures significantly more interesting than non-ambiguous ones – even though the felt fluency ratings indicate that those pictures were perceived harder.

Nevertheless, in Experiment 2c, no effect of *duration* on liking was found in combination with a not significant difference for *ambiguity* on felt fluency ratings. This was puzzling in the first instance, as at 500ms it could be that both ambiguity classes were perceived equally fluent. However, no effect was found in the 10ms condition, as well, which speaks against that suggestion. We therefore hypothesized that the perceived time difference was too obvious so that the participants identified duration the source of fluency. This identification might have led to a) an adjustment of liking ratings and b) the usage of just the temporal fluency manipulation to judge felt fluency. If the source of fluency is obvious, it has been suggested that judgments are “corrected” [4,66,98,99]. Oppenheimer [4] noted on page 238 that “however, when there is an obvious alternative cause for fluency people will spontaneously discount the fluency experience, and the effects of fluency on judgment will be diminished or reversed”. In order to test this explanation, an additional Experiment with 100 and 1000 ms presentation duration was run (N = 30). The presentation durations were chosen a) to replicate one duration condition (100ms) and compare it with the already existing data and b) to check whether effects of liking get more pronounced under longer durations than 500ms.

### Experiment 3

#### Participants

Thirty undergraduate students participated for course credit. The participants’ ages (27 female, 3 male) ranged between 20 and 38 years with a mean age of 23.53 years (SD = 4.30). All had normal or corrected to normal vision and written consent was obtained from each participant.

#### Stimuli and Design

Stimuli and Design are identical to Experiment 2a, 2b, and 2c. The presentation duration was varied on two levels (100ms versus 1000ms).

### Results Experiment 3

#### Effects on liking

**Figure 5:**
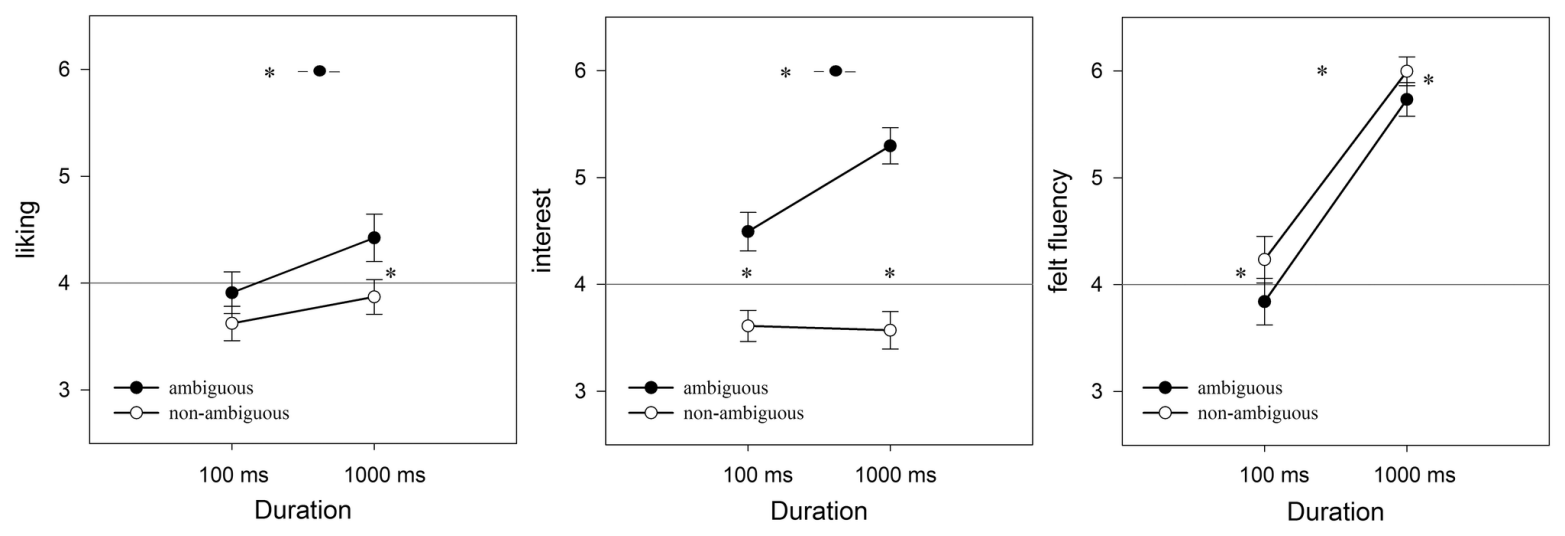
Results for liking (left), interest (in the middle), and felt fluency (right) for ambiguous (black) and non-ambiguous (white) pictures split by duration (100ms versus 1000ms).

Corresponding to the previous findings, we found significant main effects for *ambiguity*, *F*(1,29) = 10.28, *p* = .003, ηp² = .25, and *duration*, *F*(1,29) = 12.81, *p* = .001, ηp² = .31. Both factors did not significantly interact, *F*(1,29) = 1.14, *p* = .28, n.s. Simple main effects showed that the factor *duration* positively influenced only the ratings of ambiguous pictures (see Figure 5). Further, at 1000ms, ambiguous pictures were liked more than the non-ambiguous counterparts.

#### Effects on interest

An identical (to 2a, 2b, and 2c) results pattern was found for interest. Both main effects and the interaction reached significance: *ambiguity*, *F*(1,29) = 100.58, *p* <.001, ηp² = .78, *duration*, *F*(1,29) = 8.88, *p* = .006, ηp² = .24, and ambiguity × duration, *F*(1,29) = 15.16, *p* = .001, ηp² = .33.

#### Effects on felt fluency

Of most importance, effects of *ambiguity* were significantly shown in this additional experiment, *F*(1,29) = 25.96, *p* < .001, ηp² = .46. In both *duration* conditions, non-ambiguous pictures were perceived as more fluent than ambiguous ones. Moreover, *duration* showed a significant increase from 100ms to 1000ms in both ambiguity conditions, *F*(1,29) = 135.39, *p* < .001, ηp² = .81, but no interaction between the two factors was found, *F*(1,29) = 0.73, *p* = .400, n.s.

We assume that the comparison of a relatively long duration (500ms) with a brief display (10ms) presumably caused the divergent conceptual fluency effects in Experiment 2c. Furthermore, we successfully replicated our findings in one duration condition (100ms) and showed that effects of liking get more pronounced under longer durations (1000ms).

## General Discussion

The present study examined behavioral responses (classification, aesthetic judgments, felt fluency) to ambiguity (ambiguous versus non-ambiguous). In the first part, we focused on the accuracy of classification performance (ambiguous? Yes/no) at four different presentation durations (Exp. 1a: 10ms, 50ms, 100ms, 500ms) and on the speed of classification under a RT-based presentation duration. In the second part, the relationship between aesthetic appreciation and ambiguity/fluency was tested. The results of both parts will be discussed separately, leading to a final overall conclusion and outlook.

### Part A: Classifying ambiguous and non-ambiguous content

We found that the performance was above chance level even at 10ms presentation duration. Not surprisingly, the classification performance increased over the fixed presentation durations with significant differences between 10ms and 100ms as well as 10ms and 500ms (mean *d*’). In all four duration conditions mean *d*’s significantly deviated from zero suggesting that the mean performance (mean *d*’) was above chance level. These results are in line with previous findings showing that content of pictures can be processed under short (masked) presentation durations e.g. under 10ms [76] or 27ms [75]. Nevertheless, the CI of *d’* on a participant level showed that until 100ms half of the participants were not able to classify the stimuli reliably. Even though the cognitive effort to classify ambiguous picture is probably higher than classifying real-world scenes, it is surprising that even under 500ms presentation duration 4 of 16 participants performed below chance level. People differ in their speed of information processing due to differences in cognitive processing speed on a reflectivity-impulsivity continuum e.g. [100]. Therefore, such inter-personal differences might have influenced the participants’ performance.

In Experiment 1b, no RT- differences were found between ambiguous and non-ambiguous stimuli. These findings are similar to those found in studies investigating object-scene inconsistencies [53]. Võ and Henderson [53] argued that in those studies replicating the findings of Loftus and Mackworth [54], artificial saliency effects (artificial shadows based on manipulation techniques) or low complexity might have caused the pop-out effects of inconsistent objects. In our pre-studies and the main studies, participants were asked if they experienced something odd. No one mentioned picture-related facts. Thus, these results might also support the artificial saliency theory. One major difference between previous studies and the present was the task. Often visual search tasks – searching for a specific continent or inconsistent target – were used rather than our more unspecific instructions to search for any ambiguity in the stimuli. Any artistic (or art-like) stimulus might evoke a prolonged search because in such materials ambiguities arise more often. In highly controlled real-world scenes the expectation for an occurrence of an inconsistent object (and the probability that inconsistencies are present) is low; the opposite might be the case for artworks. Therefore, future studies should aim to compare our study with additional control stimuli (real world scenes) and/or use eye-tracking methods in combination with saliency toolbox to empirically check the differences or similarities to the object-scene inconsistencies discussed above. A second explanation for the found effects is related to the conceptual fluency manipulation. RT advantages were found for perceptually fluent stimuli [36]. However, Oppenheimer [4] noted that reaction time differences are not sufficient for all types of fluency and that a subjective feeling of fluency is not measured by those RT differences. Accordingly, the results for measured “felt fluency” in the present study (Part B, see below) indicate that at 10, 50, and 100ms presentation duration, the participants indeed perceived the ambiguous stimuli as more disfluent than non-ambiguous versions, in accordance with Oppenheimers’ statement. In future studies we plan to use electrophysiological methods to objectively measure effects of fluency, in addition. Some studies e.g. [7,63] showed that a specific pattern of facial muscle activation is present when fluent stimuli are presented. The subtle changes in facial muscle activation might reveal a more specific insight than RTs and be also of interest for follow-up studies of Part B.

### Part B: Appreciation of ambiguity

In Part B, a perceptual fluency manipulation (presentation duration; 10ms versus 50ms; 10ms versus 100ms; and 10ms versus 500ms) as well as a conceptual fluency manipulation (ambiguous versus non-ambiguous stimuli) were used to test whether ambiguity in the arts can be appreciated.

The *duration* hypothesis – aesthetic judgments for ambiguous and non-ambiguous judgments increase with processing fluency and thus the presentation duration - was partly confirmed by the data. In Experiment 2a (10 versus 50ms) the expected pattern for both ambiguity classes was found for liking. In Experiment 2b, post hoc tests showed only a significant increase for ambiguous but not for non-ambiguous pictures. In all experiments the very same pattern was observed for interest. In Experiment 2c (10 versus 500ms), no duration effect on liking was found but, in contrast to 2a and 2b, an effect of conceptual fluency. This supports the hypothesis that effects of ambiguity occur at longer presentation durations. Surprisingly, conceptual fluency significantly influenced the interest ratings in the favor of ambiguous pictures much earlier than liking, namely at 50, 100, and 500ms. Even though the ambiguous pictures were rated as more interesting than the non-ambiguous ones, they were subjectively more difficult to perceive at 50 and 100ms. At 500ms no significant difference was found. This finding might be due to a) the greater –and more obvious – time difference between the duration conditions (10ms versus 500ms) or b) could represent a successful, self-rewarding –personal, subjective - interpretation process. In order to test the former explanation that only the temporal fluency manipulation could have been used for the judgments of felt fluency, we ran an additional experiment (Experiment 3) which varied longer durations (100ms versus 1000ms). Our findings suggest that indeed the perceived differences in duration in Experiment 2c have caused the deviant findings. Moreover, we replicated our findings at 100ms and extend it to the 1000ms condition. Effects of liking were more pronounced than at 500ms which suggests, that effects of ambiguity emerge over time. This is also in line with previous results that prolonged viewing time allows alternative interpretation(s) [14].

Theories of aesthetic appreciation and aesthetic judgment in the arts [3] assume that the influence of variables related to earlier processing stages can be overwritten by those on later processing stages. There is some evidence for this hypothesis: Martindale, Moore, and Borkum [101] showed that semantic factors overwrote effects of complexity. Moreover, effects of “mere exposure” [102] were often not found or were weak for artworks as compared to other classes of stimuli – for a review please see 98. “Mere exposure” (“retrieval ease”) was identified as one possible fluency manipulation [34] and follows the idea, that familiar (and thus fluent) stimuli are preferred over unfamiliar ones. By combining temporal, perceptual, and conceptual fluency manipulations, we demonstrated the temporal dynamics of such complex judgments, as for example aesthetic appreciation. Under shorter presentation durations, information processing, as proposed by the model of aesthetic appreciation and aesthetic judgments, is disrupted. Consequently, those variables active on earlier processing stages like perceptual fluency mostly impact the judgmental outcome. In combination with the first part of our study, we assume that higher order semantic factors like ambiguity start overwriting effects of fluency from approximately 500 ms onwards.

We already mentioned in the short discussion section of Experiment 2a to 2c that more challenging materials might be preferred and found more interesting due to self-rewarding mechanisms [52,68]. Several studies indicate that accomplishing the challenge of perceiving an artwork goes along with activation of the rewarding centers in the brain [103–105]. Especially the very robust findings for interest show that we tap into such processes. Interest is a precondition for learning, exploration and curiosity as it draws attention to novel things and experiences [83,86]. The nice thing in the arts might be that (ambiguous) paintings or poems must not be necessarily be new (be seen or read the first time) to enhance interest. The possibility to find new interpretations each time one sees a painting [3] again might prolong the appraisal of interest as compared to real world scenes. In contrast to pleasantness which motivates to stuck with known, familiar events, the mechanism to be attracted by novel things can also have negative consequences like danger [83,86]. The “save” context of art perception but also the described characteristics of art – that there is not one solution – might be the reason why we are attracted by ambiguity in the arts.

## Conclusions

The present study empirically provides some new insights to the role of ambiguity in the arts. Even though the present set of stimuli was homogenous and therefore conclusions are limited, we showed what art-historical approaches have assumed for a long time, namely, that ambiguity is an enjoyable feature in the arts. In the past different types of ambiguity have been investigated in psychology; ambiguity and its role in aesthetic appreciation was only rarely addressed. Though artworks are a specific class of stimuli, nevertheless, stimuli like Magritte paintings definitely have features in common with e.g. linguistic stimuli, and show what visual and cognitive, lower and higher processes determine what we perceive and what we like. Ramachandran and Hirstein [52], for example, noted that surrealistic art would be closely linked to metaphorical ambiguity in language as it plays with links between semantics and vision. In the present studies, employing Magritte paintings and testing competing hypotheses, we provide empirical data beyond previous attempts to relate the engagement to (sensory) ambiguous, multi-stable images to aesthetics [106]. The advantage of the present materials was the high comparability between the two ambiguity conditions. Responses to the here used distractor images showed similar results. In future studies a broader range of controlled materials should be used to show that in contrast to ambiguity arising in social situations, ambiguity in the arts can indeed be a source of pleasure. The reason might be the save context which ensures a playful and enigmatic aspect of aesthetic experience [52]. This playful aspect of aesthetic experience can be evolutionary essential as it comprises a fictitious playground to “train” problem solving and resolving ambiguity in real-world scenarios [70].

## Supporting Information

Table S1 **List of Magritte artworks used in the present experiments (Original title, year; in alphabetic order).**
(DOCX)
